# Inhibition of the Wnt palmitoyltransferase porcupine suppresses cell growth and downregulates the Wnt/β-catenin pathway in gastric cancer

**DOI:** 10.3892/ol.2013.1256

**Published:** 2013-03-14

**Authors:** MIN-LI MO, MENG-RU LI, ZHAO CHEN, XING-WEI LIU, QING SHENG, HAI-MENG ZHOU

**Affiliations:** 1 Beijing Key Laboratory of Protein Therapeutics, School of Life Sciences, Tsinghua University, Beijing 100084;; 2 College of Life Sciences, Zhejiang Sci-Tech University, Hangzhou, Zhejiang 310018;; 3 Beijing ACCB Biotech Ltd., Beijing 100094;; 4 Zhejiang Provincial Key Laboratory of Applied Enzymology, Yangtze Delta Region Institute of Tsinghua University, Jiaxing, Zhejiang 314006, P.R. China

**Keywords:** Wnt, palmitoylation, palmitoyltransferase, porcupine, gastric cancer

## Abstract

Similarly to the Wnt protein palmitoyltransferase, porcupine (PPN) is essential to the activation of the Wnt/β-catenin signaling pathway. However, little is known about the role of PPN activity in human gastric cancer, one of the most common causes of cancer-related mortality. Real-time quantitative PCR was used to detect the expression levels of PPN in paired gastric cancer tissues. Cell proliferation, migration and invasion assays were performed following treatment using a newly developed small molecule PPN inhibitor (inhibitors of Wnt production, IWP-2) in the gastric cancer MKN28 cell line. Expression of downstream target genes and transcriptional activity of the Wnt/β-catenin signaling pathway were examined following IWP-2 treatment in MKN28. We identified that PPN was overexpressed in human gastric cancer tissue samples and cell lines. Following treatment of the gastric cancer cell line MKN28 with IWP-2, we detected that IWP-2 decreased MKN28 cell proliferation, migration and invasion, and elevated caspase 3/7 activity. Further analysis demonstrated that IWP-2 downregulated the transcriptional activity of the Wnt/β-catenin signaling pathway and downregulated the expression levels of downstream Wnt/β-catenin target genes in MKN28 cells. As current Wnt pathway-targeting strategies used for anticancer therapy have mainly focused on Wnt-receiving cells, our data shed light on the potential use of Wnt palmitoyltransferase PPN inhibitors to abrogate Wnt production in Wnt-producing cells, thus providing a potential therapeutic option for gastric cancer.

## Introduction

The Wnt signaling pathway is important in embryonic development as well as the initiation and progression of a number of types of human cancer (1–3). The Wnt/β-catenin signaling pathway, also known as the canonical Wnt signaling pathway, involves the translocation of unphosphorylated β-catenin into the nucleus. In the nucleus, β-catenin interacts with transcription factors (including TCF or LEF) to initiate the transcription of downstream target genes, including C-MYC, CCND1 and BIRC5, to regulate cell proliferation, differentiation and survival (1).

As a *Drosophila* segment polarity gene, porcupine (PPN) encodes a transmembrane protein located in the endoplasmic reticulum which is necessary for the normal processing of Wingless protein (4). Although Hofmann predicted that PPN belongs to a superfamily of membrane-bound O-acyltransferases (5), the active form of Wnt proteins were not identified to be palmitoylated on a conserved cysteine until 2003 (6). Several studies suggest that PPN-dependent palmitoylation is required for the activity and distribution of Wnt proteins (6–9).

Given that PPN acts as an essential palmitoyltransferase during the post-translational modification of Wnt proteins, a diverse library of synthetic small molecules have been screened to identify those that target Wnt-mediated cellular responses (10). One class of small molecules that inhibited the activity of PPN were named the inhibitors of Wnt production (IWP) (10). The IWP compounds share an identical core chemical structure and target the palmitoyltransferase PPN (10,11). As the majority of currently used Wnt pathway-targeting strategies have focused on Wnt-receiving cells (12,13), the potential use of IWP may provide new insights into the abrogation of aberrant Wnt signaling pathway activity in Wnt-producing cells and cancer therapy.

Gastric cancer is one of the most common causes of cancer-related mortality worldwide (14). In China, the incidence rate of gastric cancer ranked as the third highest amongst the most common cancers in 2005 (15). The initiation and progression of gastric cancer have been linked to the aberrantly activated Wnt/β-catenin signaling pathway (16–18). However, little is known on the role of PPN in gastric cancer. In this study, we first examined the expression profile of PPN in paired human gastric cancer tissue samples. We then investigated the effects of IWP on the cell growth and activity of the Wnt/β-catenin signaling pathway in the gastric cancer cell line MKN28.

## Materials and methods

### Tissue samples

Tissue samples of sixteen gastric cancer patients from local hospitals were collected following receipt of written informed consent and approval by the Tsinghua University School of Medicine Ethical Review Committee, Beijing, China. Cancerous and adjacent normal tissues of the same patient were obtained during resection and immediately snap-frozen in liquid nitrogen. Normal tissues were purchased from Biochain (Newark, CA, USA). Tissue samples were stored at −80°C prior to analysis.

### RNA extraction, reverse transcription and real-time PCR

Total RNA was isolated from tissue or culture cell samples using the RNeasy Plus kit (Qiagen Inc., Valencia, CA, USA). The concentration of RNA was examined by Nanodrop 1000 (Thermo Fisher Scientific, Wilmington, DE, USA). Reverse transcription and real-time PCR were performed as previously reported (19). The sequences of primers and probes used were: for PPN, forward: 5′-CATCCTCATCTACCTACTCAT-3′, reverse: 5′-CGCATCTTGTGCCATGTC-3′, probe: 5′-CGGTGTCTACCATGTGCATCTC-3′; for internal control (ACTB), forward: 5′-GATCATTGCTCCTCCTGAGC-3′, reverse: 5′-ACTCCTGCTTGCTGATCCAC-3′, probe: 5′-CTCGCTGTCCACCTTCCAGCAGAT-3′; for AXIN2, forward: 5′-ACATAGGTTCTGGCTATGTCTT-3′, reverse: 5′-GTCAGCGCATCACTGGATAT-3′, probe: 5′-CCACCAGCGCCAACGACAGTG-3′; for C-MYC, forward: 5′-CCACGTCTCCACACATCAG-3′, reverse: 5′-TTGGCAGCAGGATAGTCCTT-3′, probe: 5′-AACTACGCAGCGCCTCCCTCCAC-3′; for CCND1, forward: 5′-CGTCCATGCGGAAGATCGT-3′, reverse: 5′-TCCTCCTCGCACTTCTGTT-3′, probe: 5′-CTCGCAGACCTCCAGCATCCAG-3′; for BIRC5 (encoding Survivin), forward: 5′-TGGAGTCTGGGAAGGGTTGT-3′, reverse: 5′-GCTCTAACCTGCCATTGGAAC-3′, probe: 5′-TCACCCATAGCCCAGAAGCCTCA-3′. The 2^−ΔCt^ value demonstrates the relative PPN expression (relative to internal control) in cancer cell lines and normal tissues. The 2^−ΔΔCt^ value demonstrates the fold change of the relative PPN expression (relative to internal control) in cancer tissues normalized to adjacent normal tissues (20) and 2^−ΔΔCt^ >1.5 was regarded as overexpression.

### Cell culture and Wnt palmitoyltransferase inhibitor

Human gastric cancer cell lines MKN28 were obtained from the China Center for Type Culture Collection (Wuhan, China). The cells were cultured in Dulbecco’s modified Eagle’s medium supplemented with 10% fetal bovine serum. Cells were cultured at 37°C in a humid incubator with 5% CO_2_. Wnt palmitoyltransferase inhibitor (IWP-2) was purchased from Sigma Aldrich (St. Louis, MO, USA) and 30 *μ*M was used to treat the cells.

### Western blot analysis

The detailed western blot procedures were performed as previously reported (21). The primary antibodies included anti-β-actin (1:5,000; Sigma Aldrich), anti-β-catenin (1:2,000; BD Biosciences, San Jose, CA, USA), anti-Axin 2 (1:1,000; Cell Signaling Technology, Inc., Danvers, MA, USA), anti-Cyclin D1 (1:2,000; Cell Signaling Technology, Inc.), anti-c-myc (1:1,000; Cell Signaling Technology, Inc.), anti-Survivin (1:1,000; Cell Signaling Technology, Inc.). β-catenin was detected in cytosolic proteins. All other targets were detected in total proteins.

### Cell proliferation assay

CellTiter 96 Aqueous Non-Radioactive Cell Proliferation Assay kit (Promega Corporation, Madison, WI, USA) was used as previously reported (21).

### Colony formation and soft agar assays

The detailed procedures for colony formation and soft agar assays were performed as previously reported (21).

### Luciferase assay

Dual-Glo Luciferase Assay System (Promega Corporation) was used as previously reported (21). The ratio between firefly luciferase activity and renilla luciferase activity (FL/RL) was calculated to examine the TCF/LEF transcriptional activity.

### Caspase-3/7 activity assay

Caspase-Glo 3/7 Assay (Promega Corporation) was used for measuring caspase-3/7 activity according to the manufacturer’s instructions. Treated cells (100 *μ*l of 5×10^3^) were incubated with an equal volume of the assay reagent at room temperature. After 1.5 h, luminescence was measured to calculate caspase-3/7 activity.

### Transwell migration and invasion assay

Haptotaxis chambers (8-*μ*m pore size, Corning Costar, Cambridge, MA, USA) without or with 5 mg/ml matrigel (Sigma Aldrich) were used for migration and invasion assays, respectively. The experiments were repeated three times independently.

### Statistical analysis

**Figure 1:**
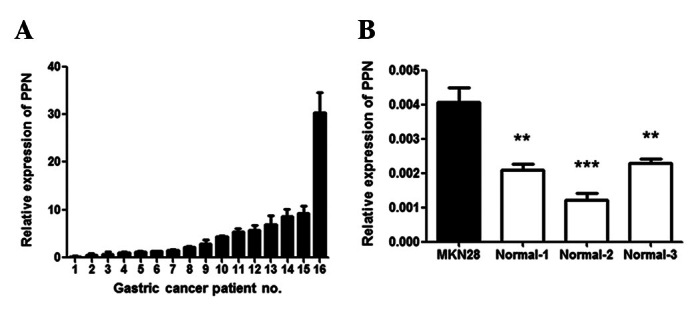
Overexpression of PPN in gastric cancer tissue samples and MKN28 cell line. (A) Real-time PCR analysis of PPN expression levels in paired gastric cancer tissue samples. The 2^−ΔΔCt^ value demonstrates the fold change of the relative PPN expression (relative to internal control) in cancer tissue normalized to adjacent normal tissue. (B) Real-time PCR analysis of PPN expression levels in gastric cancer cell line MKN28 and three normal gastric tissue samples. The 2^−ΔCt^ value demonstrates the relative PPN expression (relative to internal control). ^**^P<0.01, ^***^P<0.001. PPN, porcupine.

**Figure 2:**
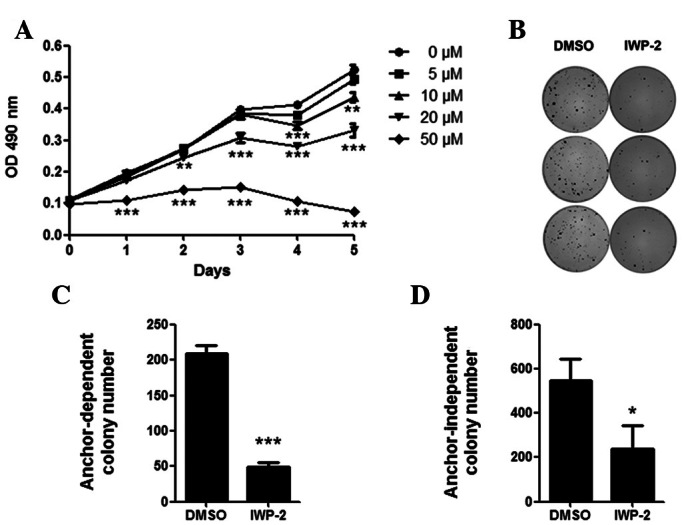
PPN inhibitor suppressed MKN28 cell growth. (A) The effect of IWP-2 at different concentrations (0–50 *μ*M) on MKN28 cell proliferation. Optical density, 490 nm. (B) Anchor-dependent colony formation images. (C) Anchor-dependent colony number counts. The cells were treated with IWP-2 for 3 weeks. (D) Anchor-independent colony number counts. The cells were treated with IWP-2 for 3 weeks. ^*^P<0.05, ^**^P<0.01 and ^***^P<0.001. PPN, porcupine; IWP, inhibitors of Wnt production.

**Figure 3:**
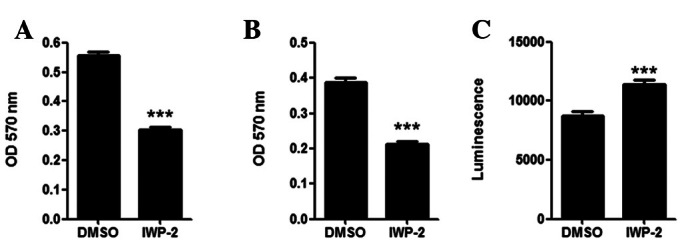
PPN inhibitor downregulated MKN28 cell migration and invasion, and induced cell apoptosis. (A) Transwell migration assay. (B) Invasion assay. (C) Caspase 3/7 activity assay. The cells were treated with IWP-2 for 3 days. Optical density, 570 nm. ^***^P<0.001. PPN, porcupine.

**Figure 4:**
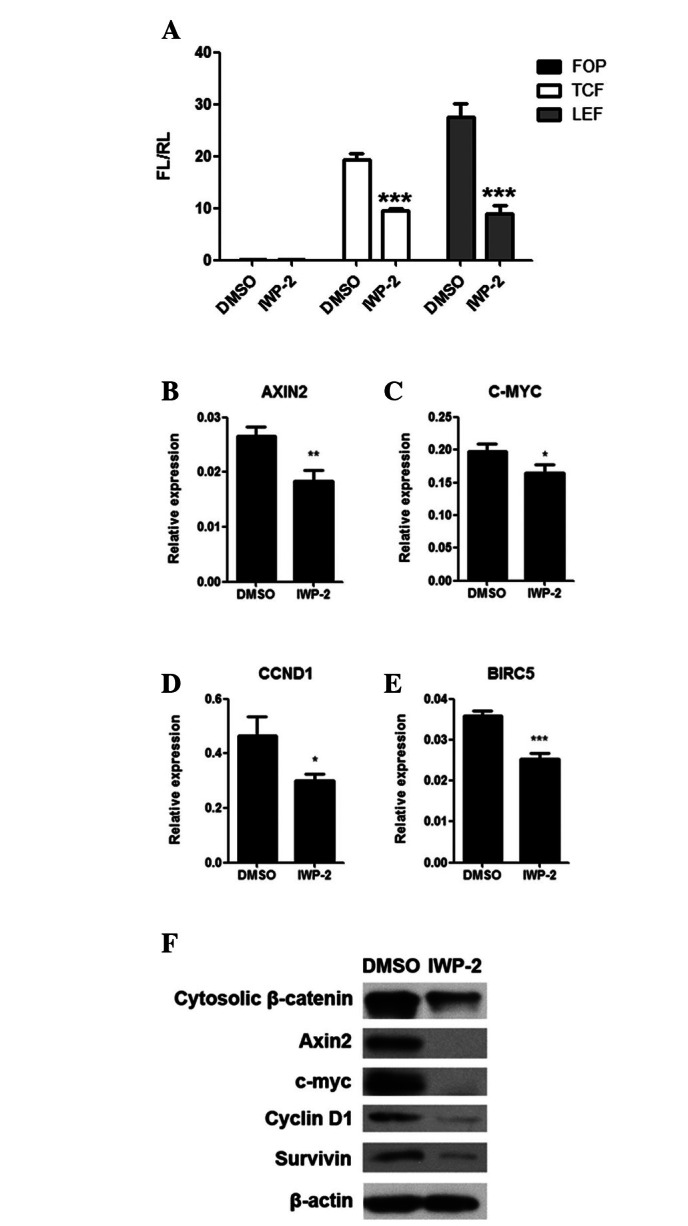
PPN inhibitor downregulated the activity of Wnt/β-catenin signaling pathway. (A) Transcriptional activity of two transcription factors (TCF and LEF). FOP was used as the negative control. (B–E) Real-time PCR analysis of downstream Wnt/β-catenin signaling pathway target genes (AXIN2, C-MYC, CCND1 and BIRC5, respectively). (F) Western blot for key regulators of the Wnt/β-catenin signaling pathway. The cells were treated with IWP-2 for 3 days. ^*^P<0.05, ^**^P<0.01 and ^***^P<0.001. PPN, porcupine; IWP, inhibitors of Wnt production.

Experimental data were analyzed using GraphPad Prism 5.0 for Windows (GraphPad Software, Inc., La Jolla, CA, USA). The differences in the relative expression levels of genes between two unpaired and paired groups were analyzed by the Student’s t-test and the Wilcoxon matched pairs test, respectively. For independently repeated experiments, mean values ± SD (error bars) are represented in Figs. 1–4. For all statistical tests, a two-tailed P<0.05 was considered to indicate a statistically significant result. P<0.05, P<0.01 and P<0.001 are represented as *, ** and ***, respectively.

## Results

### Overexpression of PPN in gastric cancer tissue samples and MKN28 cell line

PPN was overexpressed in 62.5% (10/16) of gastric cancer tissue samples compared with adjacent normal tissue samples (paired test, P<0.05; Fig. 1A). We also noted that PPN expression in the gastric cancer cell line MKN28 was significantly higher than that in three normal gastric tissue samples that we examined (P<0.05; Fig. 1B). These results indicate that PPN may be important in gastric cancer.

### PPN inhibitor suppresses MKN28 cell growth

To investigate the role of PPN as a Wnt palmitoyltransferase in gastric cancer, we used a palmitoyltransferase inhibitor (IWP-2) specific for PPN activity (10,11). Following treatment in the MKN28 cell line for four days, 10–50 *μ*M IWP-2 significantly suppressed the proliferation of MKN28 cells (P<0.05; Fig. 2A). In addition, anchor-dependent and anchor-independent colony numbers were significantly decreased following IWP-2 treatment (P<0.05; Fig. 2B–D).

### PPN inhibitor downregulates MKN28 cell migration and invasion, and elevates caspase 3/7 activity

In cell migration and invasion assays, MKN28 cells demonstrated almost 50% reduction in migration (Fig. 3A) and invasion (Fig. 3B) following IWP-2 treatment (P<0.05). IWP-2 treatment also significantly elevated the cellular caspase 3/7 activity in MKN28 cells (P<0.05), indicating that IWP-2 may induce cell apoptosis.

### PPN inhibitor downregulates activity of the Wnt/β-catenin signaling pathway

Due to the correlation of proliferation, migration and invasion of MKN28 cells with the activity of the Wnt/β-catenin signaling pathway (21), we examined the effects of IWP-2 on the Wnt/β-catenin signaling pathway in gastric cancer cells. We observed that the transcriptional activity of two transcription factors (TCF and LEF) were significantly decreased in MKN28 cells following IWP-2 treatment (P<0.05; Fig. 4A). The mRNA and protein expression levels of four downstream Wnt/β-catenin signaling pathway target genes (AXIN2, C-MYC, CCND1 and BIRC5) were also significantly downregulated in MKN28 cells following IWP-2 treatment (P<0.05; Fig. 3B–F). Furthermore, the expression levels of cytosolic β-catenin protein, a hallmark of Wnt/β-catenin signaling pathway activation, was also decreased (Fig. 3F). Taken together, these data suggest that the inhibition of PPN abrogates activity of the Wnt/β-catenin signaling pathway, providing more evidence of the importance of PPN activity in the aberrant activation of the Wnt/β-catenin signaling pathway in gastric cancer.

## Discussion

Although PPN has been demonstrated to be important in the processing and distribution of Wnt proteins (6–9), little is known with regard to its characteristics in human cancer. We previously identified that PPN was overexpressed in lung cancer (22). As gastric cancer is one of the most common causes of cancer-related mortality worldwide and in China (14,15), we aimed to study the role of PPN activity in gastric cancer. We identified that PPN was overexpressed in gastric cancer tissues and the MKN28 cell line. Notably, the Wnt/β-catenin signaling pathway was aberrantly activated in gastric cancer tissues (18) and in the MKN28 cell line (21). Therefore, our results indicate that PPN overexpression correlates with the activation status of the Wnt/β-catenin signaling pathway in gastric cancer.

We previously demonstrated that the proliferation of MKN28 cells correlated with the activity of the Wnt/β-catenin signaling pathway in a Wnt ligand-dependent manner (21), suggesting the potential influence that PPN inhibition may exert on MKN28 cell proliferation. In the present study, we demonstrated that IWP-2, a newly developed small molecule PPN inhibitor (10), inhibited anchor-dependent and anchor-independent proliferation of MKN28 cells, providing evidence that inhibiting PPN activity may be a novel strategy for suppressing gastric cancer cell proliferation.

As downstream target genes of the Wnt/β-catenin signaling pathway, CCND1 and C-MYC encode regulators of cancer cell proliferation, migration and invasion (21,23,24). Therefore, downregulation of these genes following IWP-2 treatment correlated with downregulated activity of the Wnt/β-catenin signaling pathway and inhibition of MKN28 cell proliferation, migration and invasion induced by IWP-2. In addition, downregulation of BIRC5, encoding Survivin which inhibits caspase activity and cell apoptosis (25), correlated with elevated activity of caspase 3/7 in MKN28 cells following IWP-2 treatment. These results provide further evidence for the use of PPN inhibitors to abrogate the aberrantly activated Wnt/β-catenin signaling pathway.

In summary, we demonstrated that PPN expression levels are upregulated in gastric cancer tissues. We also revealed that IWP-2, a palmitoyltransferase inhibitor specific for PPN, inhibited cell proliferation, migration and invasion, as well as inducing apoptosis in gastric cancer cells. Further analysis showed that activity of the Wnt/β-catenin signaling pathway was also downregulated by IWP-2 in these gastric cancer cells. Our data indicate that PPN activity may be critical for cell proliferation and activation of the Wnt/β-catenin signaling pathway in gastric cancer. These data also provide evidence for the viability of targeting the Wnt/β-catenin signaling pathway in Wnt-producing cells of gastric cancer and shed light on the potential use of PPN inhibitors as a therapeutic strategy for the treatment of gastric cancer.
